# Effects of Salinity on the Growth Performance and Docosahexaenoic Acid Positional Distribution in Triacylglycerols of the Newly Isolated *Schizochytrium* sp. FJ-1

**DOI:** 10.3390/md23070260

**Published:** 2025-06-23

**Authors:** Sitong Ye, Xiaonan Wang, Youcai Zhou, Xuehua Xiao, Pingying Liu, Chengdeng Chi, Peipei Sun, Mingmin Zheng, Bilian Chen, Ruoyu Mao, Yongjin He

**Affiliations:** 1 College of Life Science, Fujian Normal University, Fuzhou 350117, China; yesitong1203@163.com (S.Y.);; 2 School of Food and Biological Engineering, Fujian Polytechnic Normal University, Fuqing 350300, China; 3 Institute of Food Science and Technology, Chinese Academy of Agricultural Sciences, Beijing 100193, China; 4 National Center of Technology Innovation for Comprehensive Utilization of Saline-Alkali Land, Dongying 257091, China; 5 Feed Research Institute, Chinese Academy of Agricultural Sciences, Beijing 100081, China

**Keywords:** *Schizochytrium* sp., polyunsaturated fatty acids, docosahexaenoic acid, triacylglycerols, positional distribution

## Abstract

*Schizochytrium*-derived omega-3 polyunsaturated fatty acids (e.g., docosahexaenoic acid (DHA) and eicosapentaenoic acid (EPA)) are proven to be health-beneficial bioactive substances that have been widely applied in the pharmaceutical, nutraceutical, and food industries. In this work, the newly isolated *Schizochytrium* sp. FJ-1 strain was selected to investigate the effects of salinity on the growth performance, lipid production, DHA yield, and positional distribution of triacylglycerols (TAGs). In addition, *Schizochytrium* sp. 20888 was used as a control strain. The obtained results showed that *Schizochytrium* sp. FJ-1 could grow with a low biomass in the absence of sea salt; however, *Schizochytrium* sp. 20888 did not grow in the medium without sea salt. Moreover, *Schizochytrium* sp. FJ-1 achieved the highest biomass in 10‰ salinity, whilst *Schizochytrium* sp. 20888 attained the greatest biomass in 40‰ salinity. In terms of the total lipid content and TAG fraction percentage, *Schizochytrium* sp. FJ-1 grown in 5–20‰ salinity had high total lipid contents (57.04–60.02%), with TAGs accounting for over 90% of the lipid fraction. The highest DHA contents for total lipids (41.38%) and TAGs (40.18%) were obtained when *Schizochytrium* sp. FJ-1 was grown under 10‰ salinity conditions. Additionally, under the same culture condition, EPA contents of lipids and TAGs were significantly higher for *Schizochytrium* sp. FJ-1 compared with *Schizochytrium* sp. 20888. Furthermore, nuclear magnetic resonance analysis found that the salinity level had a distinct impact on the positional distribution of DHA in TAGs in these two *Schizochytrium* strains. *Schizochytrium* sp. FJ-1 grown under 40‰ salinity conditions produced TAGs with the greatest percentage of *sn*-2 DHA (81.24%). The percentages were higher than those found for the other groups of this microalga and *Schizochytrium* sp. 20888. Taken together, *Schizochytrium* sp. FJ-1 could be a potential candidate to produce highly valued DHA lipids or TAG bioproducts by regulating salinity.

## 1. Introduction

Docosahexaenoic acid (DHA), a type of omega-3 polyunsaturated fatty acid, exhibits anti-inflammatory properties [1], reduces blood glucose and lipid concentrations [2], enhances immune factor expression [3], and promotes the cognitive development of infants [4,5]. At present, commercial bioproducts containing DHA have been widely applied in the pharmaceutical, nutraceutical, and food industries. Currently, the primary source of DHA for human consumption is mainly derived from fish oil. However, many studies have revealed that the oil extracted from marine fish often contains harmful heavy metals (such as mercury and lead) and pesticide residues because of ocean pollution. Thus, the safety of fish oil has come into question. To sustainably provide valuable lipids rich in DHA for consumption, scientists have found that some microorganisms, such as *Schizochytrium* strains, can produce the desired lipids with DHA [6,7]. Until now, algal oil containing DHA produced by *Schizochytrium* sp. has been approved as a new resource food by the US Food and Drug Administration, European Novel Food Regulation, and the Ministry of Health of China [8]. Therefore, research using *Schizochytrium* sp. for DHA biosynthesis and production has become a hotspot in the field of DHA oil studies.

*Schizochytrium* sp. is a typical single-celled heterotrophic marine microalga. Extensive research has demonstrated that the predominant lipidic class synthesized by *Schizochytrium* strain is triacylglycerols (TAGs), accounting for over 80% of all lipidic species [9]. As reported by T. Zhang et al., the *Schizochytrium limacinum* SR31 yields 93.81% TAGs among total lipids [10]. On the other hand, recent studies have demonstrated DHA accumulation is closely related to the distribution of DHA in digested TAGs. The main reason is that DHA distributed at the *sn*-2 position of the glycerol backbone is more conducive to the generation of vital chylomicron with *sn*-2 DHA in the intestinal tract. This is subsequently deposited it into different tissues, where the physiological functions of DHA are performed [11,12,13]. To the best of our knowledge, available studies mainly focus on the growth and DHA yield of the *Schizochytrium* strain [14,15,16,17]. However, studies using the *Schizochytrium* strain to synthesize DHA distributed at the *sn*-2 position of TAGs have not been reported.

Existing studies have shown that the growth performance and DHA yield for a given *Schizochytrium* strain are largely associated with the salinity concentration of the utilized medium [18]. W. Chen et al. found that *Schizochytrium* sp. S056 cultivated in 20‰ salinity obtained the highest biomass of 34.76 g/L and the greatest DHA yield of 6.61 g/L [19]. G. Ludevese-Pascual et al. stated that *Schizochytrium* sp. LEY7 was capable of excellent growing in 15–30‰ salinity [20]. In addition, low or high salinity had negative impacts on the growth performance and DHA biosynthesis ability of the *Schizochytrium* strain due to the unsuitable salinity that generated improper osmotic pressure of fungal cell and affected absorption of nutrients for growth [19,21]. It should be noted that these reported works have not focused on the DHA distribution of *Schizochytrium*-derived TAGs affected by different levels of salinity. Thus, it is important to investigate the effect of salinity on the growth performance, DHA biosynthesis, and TAG-derived DHA distribution of a specific *Schizochytrium* strain.

On this background, our team selected a new strain of *Schizochytrium* sp. FJ-1 isolated from the Quanzhou Bay Estuarine Wetland Nature Reserve (Quanzhou, China) that can grow in a low-salt environment. To understand the DHA distribution of TAGs, the work aimed to investigate the effects of varying salinity levels on the growth performance, lipid production, and DHA biosynthesis and distribution of *Schizochytrium* sp. FJ-1. Additionally, the positional distribution of DHA in TAGs obtained from *Schizochytrium* sp. FJ-1 was analyzed using carbon-13 nuclear magnetic resonance (NMR) spectroscopy. In the addition, the common strain *Schizochytrium* sp. 20888 with a high 18s rRNA sequence similarity to *Schizochytrium* sp. FJ-1 was selected as a control strain to analyze the different features of growth performance as well as DHA biosynthesis and distribution influenced by different salinities. The obtained details in this work will provide scientific insights into the high-value DHA-enriched algal oil obtained from the *Schizochytrium* strain.

## 2. Results and Discussion

### 2.1. Molecular Identification of Schizochytrium sp. FJ-1

**Figure 1:**
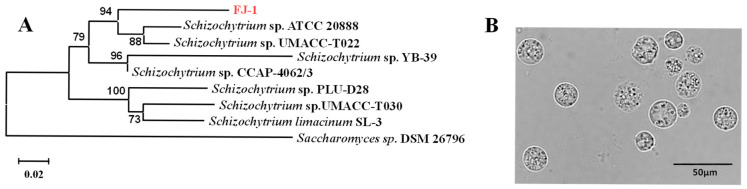
Phylogenetic tree analysis of *Schizochytrium* sp. FJ-1 based on the 18s rRNA gene (**A**) and morphological observation (**B**).

The strain *Schizochytrium* sp. FJ-1 was isolated from the estuarine wetland nature reserve of Quanzhou Bay. The 18s rRNA gene of this strain was sequenced, and the phylogenetic results showed that the isolated microalga exhibited 96% sequence similarity with *Schizochytrium* sp. UMACC-T022 (Figure 1A). In addition, the results from morphological observation (Figure 1B) found that the cell size of *Schizochytrium* sp. FJ-1 was in the range of 7 μm to 30 μm. These values were consistent with the value reported by G. Chi et al. [7]. Thus, the isolated strain FJ-1 was identified and named as *Schizochytrium* sp. FJ-1.

### 2.2. Effects of Salinity on the Growth Performance of Schizochytrium sp. FJ-1 and 20888

**Figure 2:**
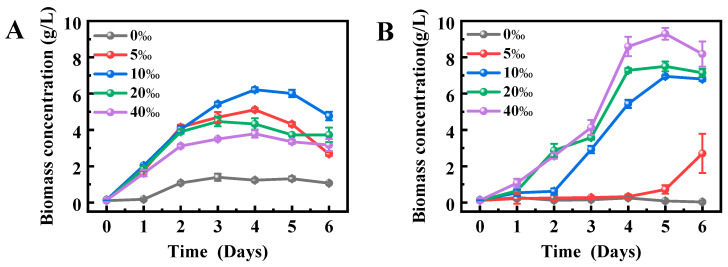
Effects of salinity on the growth performance of *Schizochytrium* sp. FJ-1 (**A**) and 20888 (**B**).

Figure 2 shows the changes in the growth of *Schizochytrium* sp. FJ-1 and 20888 under different salinity conditions. It was found that *Schizochytrium* sp. FJ-1 cultivated in the medium without sea salt had a low biomass (1.39 g/L) after 6 d (Figure 2A); however, *Schizochytrium* sp. 20888 could not grow in the absence of sea salt (Figure 2B). The possible reason was that *Schizochytrium* sp. FJ-1 was isolated from a low-salinity or sea salt-free environment in estuarine wetland nature reserve of Quanzhou Bay (Section 3.1). In this case, the isolated *Schizochytrium* sp. FJ-1 might exhibit its individual physiological characteristics to adapt to the sea salt-free condition.

Moreover, in the salinity range of 5–10‰, *Schizochytrium* sp. FJ-1 biomass rapidly increased in the early stage (0–4 days) and then distinctly decreased in the late stage (4–6 days); however, the microalgal biomass in 20–40‰ salinity was kept relatively stable (Figure 2A). The possible reason was that the different salinity levels might affect the nutrient absorption of algal cells for growth. In contrast, for *Schizochytrium* sp. 20888, microalga in 5‰ salinity had a longer lag phase (0–5 days), indicating that this salinity levels was not conducive for microalgal growth. In addition, the growth curve of *Schizochytrium* sp. 20888 in 10‰ salinity entered the stationary phase on the 5th day (Figure 2B). It was noted that the stationary phase for *Schizochytrium* sp. 20888 in 20–40‰ salinity was in the range from the 4th day to 6th day (Figure 2B). These different results from the growth curves of two *Schizochytrium* strains could be attributed to the fact that the individual metabolism features associated with growth properties of these two microalgal strains were affected by the salinity of the culture conditions.

Furthermore, the results in Figure 1A reveal that the optimal salinity for *Schizochytrium* sp. FJ-1 growth was 10‰. In addition, higher salinity (>10‰) had obvious negative impacts on the growth performance of *Schizochytrium* sp. FJ-1, as presented in Figure 2A, indicating that *Schizochytrium* sp. FJ-1 was more suitable for growth in 10‰ salinity. This level might be close to the real salinity level of its natural environment. On the other hand, the optimum salinity for *Schizochytrium* sp. 20888 growth was 40‰ (Figure 2B). The possible reason was that, among the designed salinity levels, 40‰ salinity was close to the real seawater salinity level. This value offers a stable osmotic pressure for *Schizochytrium* sp. 20888 for reproduction and nutrient absorption.

### 2.3. Effects of Salinity on Glucose Utilization by Schizochytrium sp. FJ-1 and 20888

**Figure 3:**
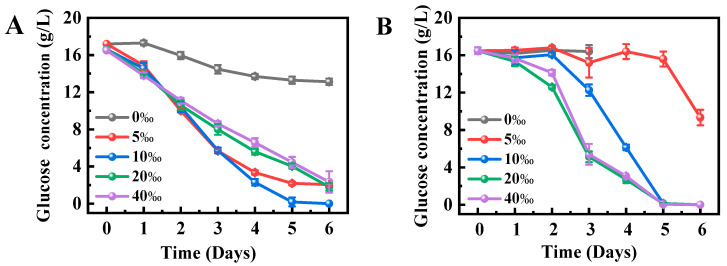
Effects of salinity on glucose utilization in *Schizochytrium* sp. FJ-1 (**A**) and 20888 (**B**).

Glucose, as the main carbon source in the culture medium, can be utilized by *Schizochytrium* sp. for growth and polyunsaturated fatty acid synthesis [22]. Figure 3A illustrates the glucose utilization of *Schizochytrium* sp. FJ-1 under different salinity levels. As depicted in Figure 3A, *Schizochytrium* sp., FJ-1 in the absence of sea salt just utilized around 3 g glucose of the fermentation medium, leading to the low biomass (Figure 2A). It was noted that *Schizochytrium* sp. FJ-1 could utilize all glucose of the medium with 10‰ salinity on the 5th day. However, microalga incompletely consumes glucose at the other salinity levels, as shown in Figure 3A. These results indicated that different salinity levels might influence the osmotic pressure of microalgal cells to perform glucose transporter activity for glucose assimilation [21].

For *Schizochytrium* sp. 20888, the algal cells under sea salt-free conditions did not absorb the glucose of the culture medium (Figure 3B). This was because *Schizochytrium* sp. 20888 cells in the absence of sea salt were in a hypotonic environment that led to cell swelling and rupture, which was consistent with the phenomenon reported by Hu et al. [23]. Additionally, an increase in salinity was conducive to absorb the glucose in the culture medium by *Schizochytrium* sp. 20888 (Figure 3B), showing that the suitable salinity for glucose utilization by *Schizochytrium* sp. 20888 was 20–40‰. Based on the results from Figure 3, it was concluded that the optimal salinity for glucose utilization was 10‰ for *Schizochytrium* sp. FJ-1 and 20–40‰ for *Schizochytrium* sp. 20888.

### 2.4. Effects of Salinity on the Total Lipid Content and TAG Fraction Percentage of Schizochytrium sp. FJ-1 and 20888

**Figure 4:**
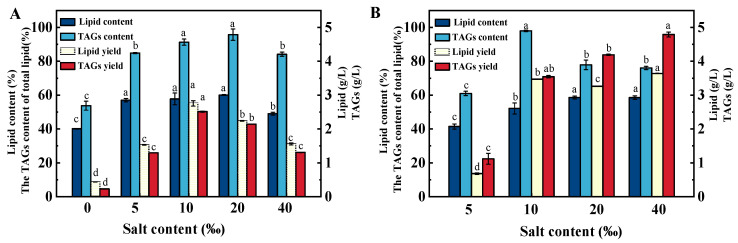
Effects of salinity on lipid and TAG content/yield for *Schizochytrium* sp. FJ-1 (**A**) and 20888 (**B**). Values are means ± SD. Values with different letters in the same column are significantly different (*p* < 0.05) (*n* = 3).

At the end of experiments, *Schizochytrium* cells were collected. The total lipid contents of the microalgal biomass are shown in Figure 4. In this study, *Schizochytrium* sp. 20888 cells could not grow under sea salt-free conditions; in this case, the total lipid content was not recorded. It was evident that *Schizochytrium* sp. FJ-1 in 5–20‰ salinity achieved the highest total lipid contents. In contrast, low or high salinity had negative impacts on lipid biosynthesis by *Schizochytrium* sp. FJ-1 (Figure 4A) due to the fact that microalgal cells grown under unsuitable salinity conditions could not assimilate glucose to offer sufficient energy for lipid biosynthesis.

Moreover, the changes in the total lipid content of *Schizochytrium* sp. 20888 are presented in Figure 4B. It was found that the increase in salinity from 5‰ to 20‰ distinctly promoted lipid synthesis in *Schizochytrium* sp. 20888 (Figure 4B). No significant differences in total lipid contents were observed in 20‰ and 40‰ salinity, as shown in Figure 4B, demonstrating that *Schizochytrium* sp. 20888 cultivated in 20–40‰ salinity exhibited the great capability for lipid biosynthesis. Furthermore, the highest lipid content (58.6%) of *Schizochytrium* sp. 20888 was very close to that of *Schizochytrium* sp. FJ-1 (60.07%) (Figure 4), showing that *Schizochytrium* sp. FJ-1 is a microorganism that can produce the desired lipid.

It is well-known that the lipids synthesized by the *Schizochytrium* strain mainly exist as TAGs. The silica column technique was employed to record the TAG fraction percentage of the total lipids extracted from *Schizochytrium*. It was found that the TAG percentage of total lipids obtained from *Schizochytrium* sp. FJ-1 sharply increased from 53.76% to 95.73% when the salinity level was in the range of 0–20‰ (Figure 4A); however, high salinity (40‰) was unfavorable for the TAGs biosynthesis by *Schizochytrium* sp. FJ-1. Similarly, *Schizochytrium* sp. 20888 grown in low- or high-salinity conditions could not attain high TAG levels among total lipids. It was noted that *Schizochytrium* sp. 20888 in 10‰ salinity achieved the highest TAG percentage (97.96%) of total lipids. Based on the obtained results, it was suggested that the salinity level might regulate the activities of key enzymes (e.g., glycerol-3-phosphate acyl-transferase (GPAT), lysophosphatidic acid acyltransferase (LPAAT), diacylglycerol acyltransferase (DGAT)) of the Kennedy pathway for TAG biosynthesis by the *Schizochytrium* strain. Additionally, previous works have demonstrated that overexpression of LPAAT and/or DGAT obviously elevated the TAG content among synthesized lipids in oleaginous microorganisms [24,25,26].

Additionally, under the optimal salinity conditions, the lipid and TAG yields of *Schizochytrium* sp. 20888 and *Schizochytrium* sp. FJ-1 were 4.19 and 2.75 g/L, respectively (Figure 4). The phenomenon was due to the fact that the lipid and TAG yields were largely related with the growth stage of the strain and the final biomass (Figure 3). Thus, it was concluded that the best salinity levels for *Schizochytrium* sp. FJ-1 and 20888 to produce the greatest lipid and TAG yields are 10‰ and 40‰, respectively.

### 2.5. Effects of Salinity on the Fatty Acid Composition of Total Lipids from Schizochytrium sp. FJ-1 and 20888

The fatty acid composition of the extracted total lipids from two *Schizochytrium* strains is presented in Table 1. As seen in Table 1, when the salinity increased from 0‰ to 10‰, the DHA content of *Schizochytrium*-derived total lipids was remarkably increased from 31.9% to 41.38% (Table 1), and DPA content significantly decreased. However, high salinity might improve the biosynthesis of specific saturated or monounsaturated fatty acids, leading to the low PUFA content for *Schizochytrium* sp. FJ-1 (Table 1). Similarly, *Schizochytrium* sp. 20888 grown in 10–20‰ salinity obtained the highest PUFA content. Low- and high-salinity levels are conducive to the production of saturated or monounsaturated fatty acid(s) by *Schizochytrium* sp. 20888. Similar phenomena were reported by An M, showing that the dehydrogenase and elongase activities of fatty acid biosynthesis and/or the polyketide synthase (PKS) pathway for a given microorganism were largely associated with salinity [27,28].

It is worth noting that *Schizochytrium* sp. FJ-1 cultivated in 10–40‰ salinity achieved 6.44–7.17% EPA. This was higher than that noted under the low-salinity condition, implying that high salinity was beneficial to modulate EPA biosynthesis pathway activity. In addition, the highest EPA content of *Schizochytrium* sp. 20888 was significantly lower than that noted for *Schizochytrium* sp. FJ-1. Additionally, the EPA content of *Schizochytrium* sp. FJ-1 was close to that of *Schizochytrium* sp. M20231041, but was comparable to the values obtained for *Schizochytrium* sp. HX-308 and S056 and *S. limacinum* SR21 (Table 2). Thus, the results on the EPA content of *Schizochytrium* sp. FJ-1 showed that this microalga could be a potential microorganism to produce the highly valued EPA.

On the other hand, DPA and DHA were the main PUFAs synthesized by two *Schizochytrium* strains, as stated in Table 1. *Schizochytrium* sp. FJ-1 grown in 10‰ salinity had the highest contents of DPA (17.92%) and DHA (41.38%); however, *Schizochytrium* sp. 20888 could achieve higher DPA and DHA contents in comparison to the results obtained for *Schizochytrium* sp. FJ-1 (Table 1). The possible reason was that these two *Schizochytrium* strains demonstrated their natural fatty acid biosynthesis metabolism (e.g., PKS pathway) toward DPA and DHA. In addition, as stated in Table 2, *Schizochytrium* sp. FJ-1 grown in 10‰ salinity obtained 17.92% DPA and 41.38% DHA. These values were comparable to those obtained for *S. limacinum* SR21, *Schizochytrium* sp. 20888, and *Schizochytrium* sp. S056, but was lower than values noted for *Schizochytrium* sp. HX-308 and *Schizochytrium* sp. S31. Some previous studies pointed out that the DPA and DHA contents of *Schizochytrium* strains were influenced by the cultivation conditions. To increase the DPA and DHA contents, further work will optimize the cultivation parameters for DPA and DHA biosynthesis by *Schizochytrium* sp. FJ-1.

### 2.6. Effects of Salinity on the Fatty Acid Composition of the TAG Fraction from Schizochytrium sp. FJ-1 and 20888

**Figure 5:**
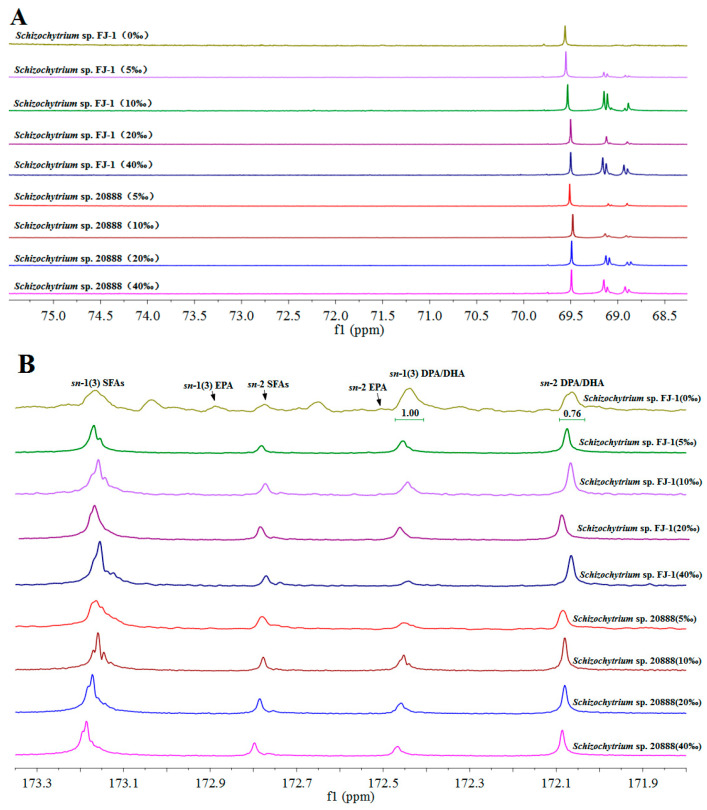
The glycerol backbone carbon regions of 68–75.5 ppm (**A**) and 171.8–173.35 ppm (**B**) for the *Schizochytrium* TAGs obtained using ^13^C NMR analysis.

The TAGs of *Schizochytrium*-derived lipids were purified using a silica column. It was found that the purified TAGs of the two *Schizochytrium* strains exhibited several chemical shifts in the range of 68.7–69.7 ppm (Figure 5A) that were consistent with the previous studies using *Schizochytrium* TAGs. The results showed that the lipids purified using the silica column were mainly comprised of TAG species.

The fatty acid composition of *Schizochytrium* TAGs is recorded in Table 3. It was clear that the TAGs of *Schizochytrium* sp. FJ-1 grown in the absence of sea salt had the lowest DHA content and the greatest palmitic acid content, as stated in Table 3. Nevertheless, *Schizochytrium* sp. FJ-1 grown in 10‰ salinity synthesized TAGs with the highest EPA, DPA, and DHA contents (Table 3). Higher salinity could significantly lower PUFA biosynthesis by *Schizochytrium* sp. FJ-1, as shown in Table 3. These results further indicated that low and high salinity levels might regulate the activities of dehydrogenase and elongase —enzymes involved in lipid metabolism—to a great extent, influencing the synthesis of PUFAs in this microalga. Moreover, except at 10‰ salinity, the EPA, DPA and DHA contents (Table 3) in *Schizochytrium* sp. FJ-1 were lower across the four groups than those in its total lipids (Table 1), implying that these fatty acids might be preferentially distributed in other lipidic species.

**Figure 6:**
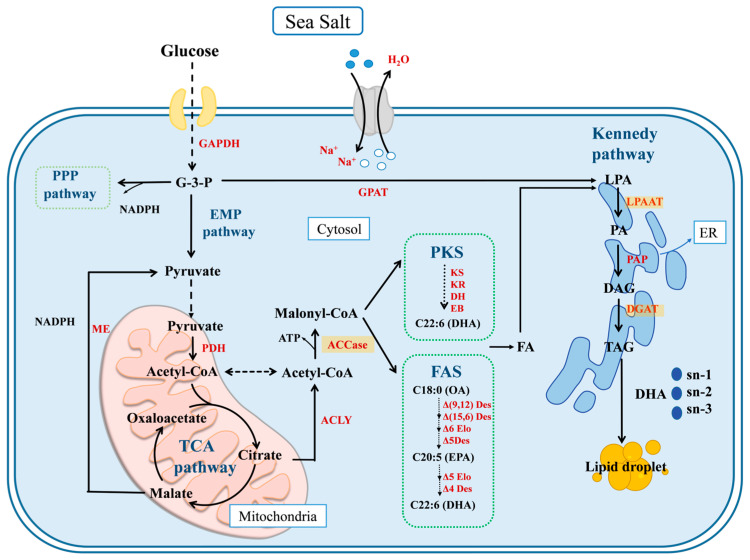
The potential mechanism for TAG biosynthesis in *Schizochytrium* cells mediated by salinity conditions (G-3-P: glyceraldehyde-3-phosphate; PKS: polyketide synthase; FAS: fatty acid synthase; GAPDH: glyceraldehyde-3-phosphate dehydrogenase; PDH: pyruvate dehydrogenase; ME: malic enzyme; ACLY: ATP-citrate lyase; Accase: acetyl-CoA carboxylase; GPAT: glycerol-3-phosphate acyltransferase; KS: ketoacyl synthase; KR: ketoreductase; DH: dehydratase; ER: enoylreductase; Des: desaturase; Elo: elongase; LPA: lysophosphatidic acid; LPAAT: lysophosphatidic acid acyltransferase; PA: phosphatidic acid; PAP: phosphatidic acid phosphatase; DAG: diacylglycerol; DGAT: diacylglycerol acyltransferase; TAG: triacylglycerol; ER: endoplasmic reticulum; *sn*-: stereospecific numbering; OA: oleic acid; EPA: eicosapentaenoic acid; DHA: docosahexaenoic acid).

Moreover, the fatty acid composition of TAGs obtained from *Schizochytrium* sp. 20888 was very close to the results for total lipids (Table 1 and Table 3). For instance, the TAGs of *Schizochytrium* sp. 20888 had 1.59% EPA, 19.32% DPA, and 50.61% DHA, and these values were very consistent with the values for total lipids (EPA, 1.64%; DPA, 20.1%; DHA, 50.89%). The results in Table 1 and Table 3 showed that the two *Schizochytrium* strains indeed utilized their natural TAG biosynthesis pathway that exhibits a preference toward fatty acid selectivity. It had been reported that the LPAAT and DGAT of the Kennedy pathway in microorganisms exhibit fatty acid selectivity and positional specificity [34] (Figure 6). The results obtained by L. L. Wayne et al. demonstrated that expression of the *Schizochytrium* LPAAT in DHA-producing Arabidopsis significantly increased the total DHA amount in seed oil and drove DHA accumulation at the *sn*-2 position of TAGs [35]. Moreover, many review articles and experimental data demonstrated that overexpression of the DGAT gene(s) could increase PUFA biosynthesis and the positional distribution of microalga-derived TAGs [26]. On the basis of the results in Table 3, it was suggested that salinity might modulate the activities of key enzymes in the Kennedy pathway, promoting the deposition of PUFAs into TAGs species by the *Schizochytrium* strain [21,24].

The ^13^C-NMR technique is a useful tool to record the positions of some fatty acid species of TAGs. The resonances of *Schizochytrium*-TAGs acyl chains (carbonyl carbons) were from 172 to 173.4 ppm as presented in Figure 5B. The resonances of SFAs (PA, etc.) chains at the *sn*-1,3 and *sn*-2 positions of TAGs were 173.25 ppm and 172.86 ppm, respectively (Figure 5B). The ∆9 fatty acyl residues (palmitoleic acid and oleic acid) were recorded at the peak of 173.24 ppm for the *sn*-1 and 3 positions and the peak of 172.83 ppm for the *sn*-2 position. The chemical shifts of EPA (∆5 fatty acyl chain) at the *sn*-1,3 and *sn*-2 positions were 172.94 ppm and 172.6 ppm, respectively. DPA (n-6, ∆4 fatty acyl chain) and DHA (*n*-3, ∆4 fatty acyl chain) were recorded in the same ^13^C-NMR spectrum (*sn*-1 and 3, 172.52 ppm; *sn*-2, 172.14 ppm). These findings agreed with the results reported by L. Shen et al. [36].

Additionally, many studies have shown that the ^13^C-NMR tool could quantify the specific fatty acids (e.g., EPA, DHA) of TAGs [37]. After the analysis of EPA and DPA/DHA among the purified TAGs, it was clear that salinity had remarkable impacts on the positional distribution of EPA and DPA/DHA among the TAGs obtained from two *Schizochytrium* strains (Figure 5B). It was noted that the TAGs obtained from the *Schizochytrium* sp. FJ-1 grown in the absence of sea salt had the lowest EPA (53.33%) and DPA/DHA (43.2%) percentages at the *sn*-2 position. In addition, the highest DPA/DHA percentage (81.24%) at the *sn*-2 position of TAGs was observed in *Schizochytrium* sp. FJ-1 cultivated in 40‰ salinity (Table 4), which was significantly greater than the values obtained at the other salinity levels. Additionally, TAGs obtained from *Schizochytrium* sp. FJ-1 grown in the presence of sea salt had higher percentages of EPA (64.55–69.23%) in comparison to the group grown in the absence of sea salt (Table 4). These results further indicated that the salinity level indeed affected the positional distribution of EPA, DPA and DHA among the TAGs synthesized by *Schizochytrium* sp. FJ-1 by regulating the fatty acid selectivity of key enzymes of the Kennedy pathway (Figure 6). Regarding *Schizochytrium* sp. 20888, the increase in salinity distinctly led an increase in the percentage of DPA/DHA distributed at the *sn*-2 position of TAGs (Table 4). However, the highest *sn*-2 EPA percentage was found for 10‰ salinity, and this results was different from the results obtained for *Schizochytrium* sp. FJ-1. The phenomenon could be due to the fact that different *Schizochytrium* strains possess distinct triacylglycerol biosynthesis pathways, with specific regulatory enzymes influencing fatty acid selectivity and positional specificity (Figure 6).

As mentioned above, the distribution of PUFAs at the *sn*-2 position of TAGs is beneficial for promoting their deposition into tissues to perform their biological functions. For *Schizochytrium* sp. FJ-1, 10‰ salinity was helpful for improving growth performance, but was not conducive for the synthesis and distribution of PUFAs at the *sn*-2 position of TAGs. To address this issue, the future work will further illustrate the potential regulatory mechanisms governed by salinity that affect the biosynthesis and positional distribution of PFUAs in *Schizochytrium* sp. FJ-1 using multi-omics techniques. These mechanisms will be leveraged to increase the growth rate and PUFA biosynthesis, thereby promoting the distribution of PUFAs at the *sn*-2 position of TAGs for high-valued lipid production by this microalga.

## 3. Materials and Methods

### 3.1. Strains of Schizochytrium

The strain *Schizochytrium* sp. FJ-1 was isolated from the Quanzhou Bay Estuarine Wetland Nature Reserve and stored in the laboratory. *Schizochytrium* sp. ATCC-20888 was kindly donated by Dr. Sun Dongzhe (College of Life Sciences, Hebei Normal University).

### 3.2. Culture Media

Seed liquid medium (g/L): glucose 20 g, yeast extract 4 g, peptone 4 g, sea salt 20 g, natural pH, trace elements 1 mL, sterilized at 115 °C for 15 min.

Fermentation medium: glucose 20 g, yeast extract 4 g, peptone 4 g, sea salt (0–40 g), natural pH, trace elements 1 mL, sterilized at 115 °C for 15 min.

Trace element stock solution: 52 mg/L ZnSO_4_, 52 mg/L MnCl_2_, 500 mg/L CaCl_2_, 100 mg/L FeSO_4_, 100 mg/L H_3_BO_3_, 480 mg/L CuSO_4_, 7.6 mg/L vitamin B_1_, 12 mg/L vitamin B_12_.

### 3.3. Effects of Different Salinity Levels on the Fermentation Performance of Schizochytrium sp. FJ-1 and 20888

*Schizochytrium* cells were incubated in 250 mL Erlenmeyer flasks containing 50 mL of seed liquid medium at 28 °C and 200 rpm for 48 h. Then, the *Schizochytrium* cells (0.1 g/L) were treated in the fermentation media containing different concentrations of sea salt (0, 5, 10, 20, and 40‰). Microalgal cells were cultivated in the dark at 28 °C and 200 rpm for 6 days. During fermentation, samples were taken every 24 h to measure the biomass and glucose consumption. At the end of experiments, the biochemical components of the algal cells were determined using the following methods.

### 3.4. Analytical Methods

#### 3.4.1. Determination of *Schizochytrium* sp. Biomass and Glucose Concentration of the Culture Medium

*Schizochytrium* sp. biomass was primarily determined using the dry weight method. One milliliter of algal solution was centrifuged at 8000 rpm for 5 min. Then, the supernatant was discarded, and the algal pellet was washed twice with ultrapure water and collected with centrifugation. The pellet was then placed on a pre-weighed glass dish and dried in an oven at 80 °C to obtain a constant weight. The microalgal biomass was calculated using the following equation:

(1) $$Biomass\;Concentration\left(\frac{g}{L}\right)=\frac{A\left(g\right)-B\left(g\right)}{The\;volume\;of\;the\;algal\;solution\;taken\left(L\right)}$$

where A is the glass dish weight containing the dried microalgal cells, and B is the glass dish weight without microalgal cells.

The glucose concentration of the culture medium was determined using the SBA-40E biosensor analyzer (Biology Institute of Shandong Academy of Sciences, Jinan, China). The measurement procedure was as follows: 1 mL of the algal solution was centrifuged at 8000 rpm for 5 min; consequently, the supernatant was collected to record the residual glucose concentration of the medium.

#### 3.4.2. Determination of Total Lipid Content of *Schizochytrium* Cells

After fermentation, the fermentation culture was centrifuged to collect the algal cells. The freeze-drying method was then employed to prepare the dry algal powder.

The total lipid content of microalgal cells was detected using the following experimental steps [38]. Briefly, 20 mg algal powder was treated with 3 mL of a chloroform–methanol solution (2:1, v: v) for 12 h. Afterward, 1 mL of distilled water was added and centrifuged at 8000 rpm for 5 min to collect the chloroform phase. The precipitate was further treated twice to collect the chloroform phase. The organic solvent of the combined chloroform sample was removed using a rotary evaporator RE-52AA (Shanghai Yarong Biochemical Instrument Factory, Shanghai, China). The total lipid content was recorded using the following equation:

(2) $$Total\;lipid\;content\left(\%\right)=\frac{C\left(mg\right)}{Algal\;powder\left(mg\right)}\times 100\%$$

where C is the weight of extracted lipids (mg).

#### 3.4.3. Determination of the TAG Fraction Percentage of Total Lipids in *Schizochytrium* sp. 

Freeze-dried *Schizochytrium* sp. powder (1 g) and chloroform–methanol solution (2:1, v:v) of 100 mL were mixed to extract total lipids. Afterward, 200 mg total lipids and 10 mL acetone were mixed thoroughly for 30 min. Then, the acetone-soluble fraction was collected. After removing the acetone by evaporation, the collected lipids were further purified using a silica column according to our reported method [39]. Subsequently, TAGs fraction was treated to remove the organic solvent. The TAG percentage of total lipids was calculated using the following equation:

(3) $$TAGs\;percentage\left(\%\right)=\frac{D\left(mg\right)}{E\left(mg\right)}\times 100\%$$

where D is the weight of TAGs (mg), and E is the weight (mg) of total lipids of the treated *Schizochytrium* stains.

#### 3.4.4. Determination of Fatty Acid Composition

The samples obtained as the total lipid and TAG fractions were methylated and quantified using a gas chromatography-flame ionization detector (GC-FID) (SCION 436-GC, Bruker, Billerica, MA, USA) equipped with an Omegawax^®^ 250 capillary column (30 m × 0.32 mm × 0.25 μm, Supelco, Bellefonte, PA, USA). Specific fatty acid profiles were identified using a mixture of 37 standards (Supelco Inc., Bellefonte, PA, USA) as well as methyl ester (HAME, 99% purity) as a standard.

#### 3.4.5. C-13 Nuclear Magnetic Resonance (^13^C-NMR) Analysis of *Schizochytrium*-Derived TAGs

Approximately 200 mg of the *Schizochytrium*-derived TAGs fraction was dissolved in 500 μL deuterated chloroform (CDCl_3_), and the solution was placed in the NMR tube. Quantitative 13-C NMR spectra were recorded on a Bruker Avance 600 MHZ spectrometer (Bruker Co. Ltd., Fällanden, Switzerland). All fatty acid peaks in the range of 171.9–173.4 ppm were integrated using MestReNova 10 software based on previous works. The percentages of EPA, DPA, and DHA distributed at the *sn*-1 (3) and 2 positions as determined using ^13^C-NMR were estimated with the following equations:

(4) $$sn\text{-}1\left(3\right)FA\;percentage\left(\%\right)=\frac{\;sn\text{-}1\left(3\right)FA}{sn\text{-}1\left(3\right)FA+sn\text{-}2\;FA}\times 100\%$$

(5) $$sn\text{-}2\;FA\;percentage\left(\%\right)=\frac{\;sn\text{-}2\;FA\;}{sn\text{-}1\left(3\right)FA+sn\text{-}2\;FA}\times 100\%$$

### 3.5. Data Processing and Statistical Analysis

All data in this experiment were processed from three repeated trials. Analysis and processing were performed using Excel 2016 and Origin 2021 software and shown as the mean (*n* = 3) ± the standard deviation (±SD). The experimental data were subjected to one-way analysis of variance (ANOVA) as implemented in the GraphPad prism 8 statistics platform. Before performing ANOVA, the assumptions of normality and homogeneity of variances were verified and satisfied. Tukey simultaneous tests were conducted to determine the statistical differences between treatments. In order to ascertain whether the observed variations in lipid and TAG contents/yield or the fatty acid composition of *Schizochytrium* total lipids/TAGs under different salinity conditions were statistically significant, probability (*p*) values were determined. A 95% confidence level (*p* < 0.05) was applied for all analyses.

## 4. Conclusions

In this work, the newly isolated *Schizochytrium* sp. FJ-1 strain was selected to investigate the influences of salinity on the growth performance, lipid production, DHA yield, and positional distribution of TAGs. The obtained results found that microalga could grow in the absence of sea salt; however, the best growth performance was observed at 10‰ salinity. Additionally, *Schizochytrium* sp. FJ-1 grown in 10‰ salinity achieved the highest lipid, TAG, and DHA yields. Moreover, high salinity (40‰) was beneficial for the accumulation of DHA at the *sn*-2 position of TAGs in *Schizochytrium* sp. FJ-1, indicating that salinity might regulate the TAG biosynthesis pathway and influence the positional distribution of DHA. Based on these results, it was concluded that the newly isolated *Schizochytrium* sp. FJ-1 could represent a promising candidate for the highly valued TAG bioproduct with high DHA levels at the ideal position by regulating salinity.

## Figures and Tables

**Table 1:** The fatty acid composition of lipids from two *Schizochytrium* strains cultivated under different salinity conditions.

Fatty Acid	Salinity Concentration (‰)								
	*Schizochytrium* sp. FJ-1					*Schizochytrium* sp. 20888			
	0	5	10	20	40	5	10	20	40
C12:0	6.35 ± 2.32 ^B^	4.83 ± 1.17 ^B^	5.11 ± 0.59 ^B^	6.63 ± 0.23 ^B^	16.68 ± 1.83 ^A^	-	-	-	-
C14:0	1.02 ± 0.06	0.73 ± 0.17	0.59 ± 0.15	0.85 ± 0.12	1.47 ± 0.34	2.69 ± 0.85 ^B^	2.65 ± 0.25 ^B^	3.28 ± 0.22 ^B^	6.66 ± 0.08 ^A^
C15:0	1.78 ± 0.47 ^C^	10.59 ± 1.90 ^B^	8.76 ± 1.66 ^B^	16.57 ± 1.26 ^A^	11.82 ± 1.64 ^B^	9.58 ± 1.40 ^A^	5.50 ± 0.22 ^B^	3.80 ± 0.11 ^C^	1.52 ± 0.04 ^C^
C16:0	15.69 ± 1.81 ^A^	16.49 ± 2.24 ^A^	8.43 ± 0.07 ^B^	9.84 ± 0.68 ^B^	7.82 ± 0.51 ^B^	28.49 ± 5.42 ^A^	15.48 ± 1.01 ^C^	15.30 ± 0.74 ^C^	24.30 ± 0.59 ^B^
C16:1	0.64 ± 0.16 ^B^	1.88 ± 0.25 ^B^	3.05 ± 0.68 ^B^	3.44 ± 0.63 ^B^	10.65 ± 1.77 ^A^	0.90 ± 0.28 ^A^	0.43 ± 0.05 ^B^	0.29 ± 0.07 ^B^	0.61 ± 0.03 ^AB^
C17:0	0.79 ± 0.20 ^C^	8.14 ± 1.30 ^A^	4.63 ± 0.27 ^B^	6.24 ± 0.62 ^AB^	4.47 ± 0.75 ^B^	4.10 ± 0.70 ^A^	2.26 ± 0.06 ^A^	1.55 ± 0.06 ^A^	0.69 ± 0.10 ^B^
C18:0	9.64 ± 0.52 ^A^	1.85 ± 0.21 ^B^	1.56 ± 0.44 ^B^	1.98 ± 0.26 ^B^	1.90 ± 0.46 ^B^	1.34 ± 0.16	0.90 ± 0.03	0.90 ± 0.11	0.92 ± 0.16
C18:1	6.73 ± 2.70 ^A^	1.06 ± 0.64 ^B^	1.54 ± 1.09 ^B^	0.81 ± 0.65 ^B^	0.72 ± 0.07 ^B^	1.80 ± 0.71 ^A^	0.16 ± 0.01 ^B^	0.10 ± 0.01 ^B^	0.16 ± 0.01 ^B^
EPA	4.36 ± 2.14 ^B^	2.75 ± 0.16 ^B^	7.05 ± 0.76 ^A^	6.44 ± 0.56 ^A^	7.17 ± 0.40 ^A^	0.55 ± 0.06 ^B^	1.64 ± 0.10 ^A^	1.83 ± 0.20 ^AB^	0.91 ± 0.02 ^B^
DPA	21.12 ± 1.69 ^A^	17.04 ± 1.28 ^A^	17.92 ± 0.52 ^A^	13.83 ± 1.22 ^B^	11.16 ± 0.13 ^C^	12.89 ± 1.59 ^C^	20.10 ± 0.15 ^A^	19.64 ± 0.23 ^AB^	16.41 ± 0.20 ^B^
DHA	31.90 ± 3.56 ^C^	34.64 ± 5.57 ^B^	41.38 ± 0.85 ^A^	33.37 ± 1.55 ^B^	26.15 ± 1.15 ^C^	37.63 ± 3.77 ^C^	50.89 ± 0.81 ^A^	53.31 ± 1.03 ^A^	47.79 ± 0.63 ^B^
ΣSAFs	35.27 ± 0.69 ^A^	42.63 ± 0.43^A^	29.07 ± 0.54 ^C^	43.28 ± 0.89 ^A^	44.15 ± 0.91 ^A^	43.69 ± 0.19 ^A^	26.78 ± 1.02 ^C^	24.84 ± 1.46 ^C^	34.08 ± 0.56 ^B^
ΣMUFAs	7.37 ± 2.83 ^B^	2.94 ± 0.98 ^C^	4.59 ± 0.77 ^B^	4.52 ± 1.51 ^B^	11.37 ± 1.80 ^A^	2.13 ± 0.14 ^A^	0.59 ± 0.04 ^B^	0.38 ± 0.14 ^B^	0.76 ± 0.03 ^B^
ΣPUFAs	57.37 ± 0.41 ^B^	54.43 ± 0.56 ^C^	66.35 ± 2.12 ^A^	52.21 ± 0.62 ^B^	44.48 ± 0.90 ^C^	54.13 ± 0.39 ^C^	72.63 ± 1.07 ^A^	74.78 ± 1.56 ^A^	65.20 ± 0.56 ^B^

ΣSAFs, saturated fatty acids; ΣMUFAs, monounsaturated fatty acids; ΣPUFAs, polyunsaturated fatty acids. Values are means ± SD. Values with different letters in the same column are significantly different (*p* < 0.05) (*n* = 3).

**Table 2:** EPA, DPA, and DHA production capacities of different strains of *Schizochytrium* sp.

Strain	Total Lipid (%)	EPA (%)	DPA (%)	DHA (%)	Ref.
*S. limacinum* SR21	41.33	0.71	7.63	36.65	[29]
*S. limacinum* B4D1	55.98	0.42	7.71	38.70	[30]
*Schizochytrium* sp. HX-308	50.82	0.80	18.10	48.19	[31]
*Schizochytrium* sp. S056	47.27	0.67	7.25	40.23	[19]
*Schizochytrium* sp. M20231041	60.70	7.20	21.18	33.41	[32]
*Schizochytrium* sp. S31 (ATCC 20888)	51.70	2.82	19.95	50.43	[33]
*Schizochytrium* sp. FJ-1	57.81	7.05	17.92	41.38	This work

**Table 3:** The fatty acid composition of *Schizochytrium* TAGs under different salinity conditions.

Fatty Acid	Salinity Concentration (‰)								
	*Schizochytrium* sp. FJ-1					*Schizochytrium* sp. 20888			
	0	5	10	20	40	5	10	20	40
C12:0	1.59 ± 0.25	0.78 ± 0.29	0.64 ± 0.19	0.81 ± 0.26	1.36 ± 1.18	-	-	-	-
C14:0	1.46 ± 0.01	0.69 ± 0.16	0.9 ± 0.16	1.56 ± 0.01	1.37 ± 0.55	2.55 ± 0.73 ^B^	3.01 ± 0.40 ^A^	3.88 ± 0.34 ^A^	6.81 ± 0.83 ^A^
C15:0	1.84 ± 0.63 ^B^	7.57 ± 1.13 ^C^	12.53 ± 2.29 ^B^	24.43 ± 1.22 ^A^	15.54 ± 0.26 ^B^	8.99 ± 1.61 ^A^	5.51 ± 0.75 ^AB^	4.38 ± 0.19 ^B^	1.48 ± 0.2 ^C^
C16:0	25.41 ± 1.68 ^A^	20.51 ± 3.14 ^AB^	11.53 ± 0.61 ^C^	14.39 ± 3.32 ^B^	16.89 ± 0.84 ^B^	27.61 ± 5.01 ^A^	16.23 ± 2.41 ^B^	18.27 ± 0.84 ^B^	23.9 ± 2.49 ^A^
C16:1	3.67 ± 2.36 ^B^	8.35 ± 6.67 ^AB^	3.17 ± 0.75 ^B^	2.66 ± 2.02 ^C^	13.20 ± 2.32 ^A^	0.98 ± 0.17	0.57 ± 0.21	0.50 ± 0.07	0.73 ± 0.15
C17:0	0.91 ± 0.22 ^B^	6.76 ± 1.02 ^A^	6.18 ± 0.75 ^A^	9.08 ± 0.6 ^A^	5.90 ± 1.19 ^A^	3.69 ± 0.78 ^A^	2.15 ± 0.34 ^AB^	1.72 ± 0.11 ^B^	0.74 ± 0.10 ^B^
C18:0	20.58 ± 1.77 ^A^	6.22 ± 1.9 ^B^	2.6 ± 0.23 ^C^	3.41 ± 2.07 ^B^	7.51 ± 0.15 ^B^	1.79 ± 0.60	0.90 ± 0.16	1.17 ± 0.17	0.87 ± 0.16
C18:1	2.86 ± 0.45 ^A^	0.46 ± 0.19 ^B^	0.34 ± 0.11 ^B^	0.25 ± 0.16 ^B^	0.29 ± 0.13 ^B^	1.83 ± 0.65 ^A^	0.12 ± 0.01 ^B^	0.14 ± 0.05 ^B^	0.17 ± 0.01 ^B^
EPA	2.99 ± 0.04 ^B^	1.99 ± 0.42 ^B^	4.88 ± 0.8 ^A^	3.23 ± 0.23 ^B^	3.50 ± 0.25 ^B^	0.58 ± 0.05	1.59 ± 0.26	1.75 ± 0.21	0.90 ± 0.09
DPA	15.23 ± 0.11 ^A^	13.88 ± 2.86 ^A^	17.06 ± 0.67 ^A^	12.31 ± 2.51 ^AB^	10.18 ± 2.01 ^B^	12.80 ± 1.30 ^B^	19.32 ± 0.53 ^A^	18.72 ± 0.11 ^A^	16.17 ± 0.65 ^A^
DHA	23.62 ± 0.19 ^C^	32.79 ± 7.74 ^B^	40.18 ± 1.6 ^A^	27.94 ± 6.59 ^BC^	24.27 ± 2.73 ^C^	39.20 ± 3.50 ^B^	50.61 ± 3.50 ^A^	49.46 ± 1.03 ^A^	48.23 ± 3.17 ^A^
ΣSAFs	51.63 ± 0.45 ^AB^	42.53 ± 0.42 ^C^	37.37 ± 0.8 ^C^	53.61 ± 0.23 ^A^	48.56 ± 1.31 ^B^	44.61 ± 0.13 ^A^	27.79 ± 0.34 ^B^	29.43 ± 0.21 ^B^	33.8 ± 0.07 ^B^
ΣMUFAs	6.53 ± 0.67 ^B^	8.81 ± 0.75 ^AB^	0.51 ± 1.02 ^C^	2.91 ± 0.60 ^C^	13.49 ± 0.70 ^A^	2.81 ± 1.03	0.69 ± 0.16	0.64 ± 0.53	0.9 ± 0.11
ΣPUFAs	41.84 ± 0.22 ^B^	48.66 ± 1.9 ^B^	62.12 ± 0.75 ^A^	43.48 ± 2.14 ^B^	37.95 ± 0.67 ^C^	52.58 ± 0.13 ^C^	71.52 ± 2.48 ^A^	69.93 ± 0.16 ^A^	65.3 ± 1.23 ^B^

ΣSAFs, saturated fatty acids; ΣMUFAs, monounsaturated fatty acids; ΣPUFAs, polyunsaturated fatty acids. Values are means ± SD. Values with different letters in the same column are significantly different (*p* < 0.05) (*n* = 3).

**Table 4:** The percentage of EPA and DPA/DHA distributed at the *sn*-1,3 and 2 positions of TAGs derived from *Schizochytrium* sp. FJ-1 and 20888.

*Schizochytrium* Strain	EPA and DPA/DHA at the *sn*-1,3 Positions		EPA and DPA/DHA at the *sn*-2 Position	
	EPA	DPA/DHA	EPA	DPA/DHA
*Schizochytrium* sp. FJ-1				
0‰ salinity	46.67 ± 3.53	56.80 ± 3.08	53.33 ± 2.86	43.20 ± 2.78
5‰ salinity	30.76 ± 2.67	35.34 ± 2.77	69.23 ± 4.32	64.66 ± 2.58
10‰ salinity	32.33 ± 2.82	36.76 ± 2.31	67.67 ± 2.55	63.24 ± 4.02
20‰ salinity	31.58 ± 2.64	37.04 ± 2.69	68.42 ± 3.58	62.96 ± 2.96
40‰ salinity	35.45 ± 3.05	18.76 ± 1.58	64.55 ± 3.06	81.24 ± 4.05
*Schizochytrium* sp. 20888				
5‰ salinity	57.69 ± 2.56	38.46 ± 2.47	42.31 ± 1.96	61.54 ± 2.66
10‰ salinity	32.08 ± 2.53	35.46 ± 1.98	67.92 ± 2.55	64.54 ± 2.07
20‰ salinity	51.10 ± 2.66	32.05 ± 2.38	48.90 ± 2.75	67.95 ± 3.81
40‰ salinity	48.25 ± 2.96	31.15 ± 1.63	51.75 ± 2.82	68.84 ± 3.02

## Data Availability

The original contributions presented in the study are included in the article; further inquiries can be directed to the corresponding authors.

## References

[1] Djuricic I., Calder P.C. (2021). Beneficial Outcomes of Omega-6 and Omega-3 Polyunsaturated Fatty Acids on Human Health: An Update for 2021. Nutrients.

[2] Omachi D.O., Aryee A.N.A., Onuh J.O. (2024). Functional Lipids and Cardiovascular Disease Reduction: A Concise Review. Nutrients.

[3] Barta D.G., Coman V., Vodnar D.C. (2021). Microalgae as sources of omega-3 polyunsaturated fatty acids: Biotechnological aspects. Algal Res..

[4] Fleith M., Clandinin M.T. (2005). Dietary PUFA for Preterm and Term Infants: Review of Clinical Studies. Crit. Rev. Food Sci. Nutr..

[5] Derbyshire E.J., Birch C.S., Bonwick G.A., English A., Metcalfe P., Li W. (2024). Optimal omegas—Barriers and novel methods to narrow omega-3 gaps. A narrative review. Front. Nutr..

[6] Sehl A., Caderby E., Bouhouda S., Rébeillé F., Griffiths H., Da Rocha Gomes S. (2022). How do algae oils change the omega-3 polyunsaturated fatty acids market?. OCL.

[7] Chi G., Xu Y., Cao X., Li Z., Cao M., Chisti Y., He N. (2022). Production of polyunsaturated fatty acids by *Schizochytrium* (*Aurantiochytrium*) spp.. Biotechnol. Adv..

[8] Turck D., Bohn T., Castenmiller J., De Henauw S., Hirsch-Ernst K.I., Maciuk A., Mangelsdorf I., McArdle H.J., Naska A., EFSA Panel on Nutrition, Novel Foods and Food Allergens (NDA) (2023). Safety of oil from *Schizochytrium* sp. (strain CABIO-A-2) for use in infant and follow-on formula as a novel food pursuant to Regulation (EU) 2015/2283. EFSA J..

[9] Puri M., Gupta A., Sahni S. (2023). *Schizochytrium* sp.. Trends. Microbiol..

[10] Zhang T., Lou F., Tao G., Liu R., Chang M., Jin Q., Wang X. (2016). Composition and Structure of Single Cell Oil Produced by *Schizochytrium* limacinum SR31. J. Am. Oil Chem. Soc..

[11] Li F., Yibing N., Yiren Z., Huidong H., Qingbin Y., Xingguo W., Wei W. (2025). Positional distribution of DHA in triacylglycerols: Natural sources, synthetic routes, and nutritional properties. Crit. Rev. Food Sci. Nutr..

[12] Jin J., Jin Q., Wang X., Akoh C.C. (2020). High *Sn*-2 Docosahexaenoic Acid Lipids for Brain Benefits, and Their Enzymatic Syntheses: A Review. Engineering.

[13] Christensen M.S., Høy C.E., Becker C.C., Redgrave T.G. (1995). Intestinal absorption and lymphatic transport of eicosapentaenoic (EPA), docosahexaenoic (DHA), and decanoic acids: Dependence on intramolecular triacylglycerol structure. Am. J. Clin. Nutr..

[14] Wang Q., Han W., Jin W., Gao S., Zhou X. (2021). Docosahexaenoic acid production by *Schizochytrium* sp.: Review and prospect. Food Biotechnol..

[15] Sahin D., Tas E., Altindag U.H. (2018). Enhancement of docosahexaenoic acid (DHA) production from *Schizochytrium* sp. S31 using different growth medium conditions. AMB Express..

[16] Półbrat T., Konkol D., Korczyński M. (2021). Optimization of docosahexaenoic acid production by *Schizochytrium* sp.—A review. Biocatal. Agric. Biotechnol..

[17] Sun L., Ren L., Zhuang X., Ji X., Yan J., Huang H. (2014). Differential effects of nutrient limitations on biochemical constituents and docosahexaenoic acid production of *Schizochytrium* sp.. Bioresour. Technol..

[18] Gao M., Song X., Feng Y., Li W., Cui Q. (2013). Isolation and characterization of Aurantiochytrium species: High docosahexaenoic acid (DHA) production by the newly isolated microalga, *Aurantiochytrium* sp. SD116. J. Oleo Sci..

[19] Chen W., Zhou P., Zhu Y., Xie C., Ma L., Wang X., Bao Z., Yu L. (2016). Improvement in the docosahexaenoic acid production of *Schizochytrium* sp. S056 by replacement of sea salt. Bioprocess Biosyst. Eng..

[20] Ludevese-Pascual G., Dela Peña M., Tornalejo J. (2016). Biomass production, proximate composition and fatty acid profile of the local marine thraustochytrid isolate, *Schizochytrium* sp. LEY 7 using low-cost substrates at optimum culture conditions. Aquac. Res..

[21] Jiang J.Y., Zhu S., Zhang Y., Sun X., Hu X., Huang H., Ren L.J. (2019). Integration of lipidomic and transcriptomic profiles reveals novel genes and regulatory mechanisms of *Schizochytrium* sp. in response to salt stress. Bioresour. Technol..

[22] Wu S.-T., Yu S.-T., Lin L.-P. (2005). Effect of culture conditions on docosahexaenoic acid production by *Schizochytrium* sp. S31. Process Biochem..

[23] Hu X.-C., Ren L.-J., Chen S.-L., Zhang L., Ji X.-J., Huang H. (2015). The roles of different salts and a novel osmotic pressure control strategy for improvement of DHA production by *Schizochytrium* sp.. Bioprocess Biosyst. Eng..

[24] Dong L., Wang F., Chen L., Zhang W. (2023). Metabolomic analysis reveals the responses of docosahexaenoic-acid-producing *Schizochytrium* under hyposalinity conditions. Algal Res..

[25] Wang W., Xue Y., Li B., Sheng X., Shi Y., Zou Q., Li J., Li T., Wang X., Xue J. (2025). Effect of peroxisome proliferation and salt stress on enhancing the potential of microalgae as biodiesel feedstock. Renew. Sustain. Energy Rev..

[26] Wang X., Liu S.-F., Li R.-Y., Yang W.-D., Liu J.-S., Lin C.S.K., Balamurugan S., Li H.-Y. (2020). TAG pathway engineering via GPAT2 concurrently potentiates abiotic stress tolerance and oleaginicity in *Phaeodactylum tricornutum*. Biotechnol. Biofuels.

[27] An M., Mou S., Zhang X., Zheng Z., Ye N., Wang D., Zhang W., Miao J. (2013). Expression of fatty acid desaturase genes and fatty acid accumulation in *Chlamydomonas* sp. ICE-L under salt stress. Bioresour. Technol..

[28] Atikij T., Syaputri Y., Iwahashi H., Praneenararat T., Sirisattha S., Kageyama H., Waditee-Sirisattha R. (2019). Enhanced lipid production and molecular dynamics under salinity stress in green microalga *Chlamydomonas reinhardtii* (137C). Mar. Drugs.

[29] Zhu L., Zhang X., Ji L., Song X., Kuang C. (2007). Changes of lipid content and fatty acid composition of *Schizochytrium limacinum* in response to different temperatures and salinities. Process Biochem..

[30] Chen L., Liu X., Li C., Li H., Chen W., Li D. (2022). Transcriptome analyses reveal the DHA enhancement mechanism in *Schizochytrium* limacinum LD11 mutant. Algal Res..

[31] Zhang Z.-X., Wu H.-X., Lin Y.-C., Xu Y.-S., Ma W., Sun X.-M., Huang H. (2024). Polyketide synthase acyltransferase domain swapping for enhanced epa recognition and efficient coproduction of EPA and DHA in *Schizochytrium* sp.. J. Agric. Food Chem..

[32] Ou Y., Qin Y., Feng S., Yang H. (2024). Dual stress factors adaptive evolution for high EPA production in *Schizochytrium* sp. and metabolomics mechanism analysis. Bioprocess Biosyst. Eng..

[33] Chang M., Zhang T., Li L., Lou F., Ma M., Liu R., Jin Q., Wang X. (2021). Choreography of multiple omics reveals the mechanism of lipid turnover in *Schizochytrium* sp. S31. Algal Res..

[34] Sutton G.C., Quinn P.J., Russell N.J. (1990). The effect of salinity on the composition of fatty acid double-bond isomers and *sn*-1/*sn*-2 positional distribution in membrane phospholipids of a moderately halophilic Eubacterium. Curr. Microbiol..

[35] Wayne L.L., Gachotte D.J., Graupner P.R., Adelfinskaya Y., McCaskill D.G., Metz J.G., Zirkle R., Walsh T.A. (2021). Plant and algal lysophosphatidic acid acyltransferases increase docosahexaenoic acid accumulation at the *sn*-2 position of triacylglycerol in transgenic Arabidopsis seed oil. PLoS ONE.

[36] Shen L., Li F., Jiang C., Cao X., Jin J., Wang X., Wei W. (2024). Comparative analysis of DHA positional distribution and triacylglycerol molecular species in algal oil (*Schizochytrium* sp.) from different oil processing. Food Biosci..

[37] Zhao Y., Chang L., Yang B., Zhao J., Chen W., Chen H. (2025). Dynamic Changes in Lipid Composition Reveal the Mechanism of Arachidonic Acid Accumulation in *Mortierella alpina*. Food Biosci..

[38] Zhang J., He Y., Luo M., Chen F. (2020). Utilization of enzymatic cell disruption hydrolysate of *Chlorella pyrenoidosa* as potential carbon source in algae mixotrophic cultivation. Algal Res..

[39] He Y., Huang Z., Zhong C., Guo Z., Chen B. (2019). Pressurized liquid extraction with ethanol as a green and efficient technology to lipid extraction of Isochrysis biomass. Bioresour. Technol..

